# Urogenital Mycoplasmas and Human Papilloma Virus in Hemodialysed Women

**DOI:** 10.1155/2013/659204

**Published:** 2013-12-02

**Authors:** Alicja Ekiel, Bronisława Pietrzak, Barbara Wiechuła, Małgorzata Aptekorz, Natalia Mazanowska, Dominika Rady, Paweł Kamiński, Gayane Martirosian

**Affiliations:** ^1^Department of Medical Microbiology, Medical University of Silesia, Medyków 18, 40-752 Katowice, Poland; ^2^Department of Obstetrics and Gynecology, Medical University of Warsaw, Starynkiewicza 1/3, 02-015 Warsaw, Poland; ^3^Department of Histology and Embryology, Medical University of Warsaw, Chalubińskiego 5, 02-004 Warszawa, Poland

## Abstract

Bacterial infections, especially endogenous, are the frequent complications among hemodialyzed and renal transplant patients. In this study we assumed the prevalence of urogenital mycoplasmas and HPV among hemodialysed women. We examined 32 hemodialysed women aged 20–48 (mean 35.6 ± 8.23) and 100 healthy controls of the same ages. Two swabs were collected for detection of mycoplasmas and HPV. Culture of *Ureaplasma* spp. and *M. hominis* was performed using Mycoplasma IST2 (bioMérieux, France), Identificaton of *U. parvum* and *U. urealyticum* was performed by Kong. Primers described by Jensen were used for *M. genitalium*. For detection of high-risk HPV types Amplicor HPV (Roche Molecular System, CA) was used. Prevalence of urogenital mycoplasmas in the hemodialysed women (53.1%) was significantly higher (*P* = 0.0059), compared with controls (25%). In both groups, *U. parvum* was the most frequently isolated. Cooccurrence of urogenital mycoplasmas was shown in 75% of the HPV-positive hemodialysed women and in 30.4% of HPV-positive controls (*P* = 0.0461). Cooccurrence of urogenital mycoplasmas with HPV was significantly higher in hemodialysed women. The need to take into account these microorganisms in routine diagnostic, especially for hemodialysed patients, was demonstrated. Further studies to demonstrate the role of this cooccurrence in etiopathogenesis of infection in hemodialysed patients are required.

## 1. Introduction

Bacterial infections are the frequent complications observed in hemodialyzed and renal transplant patients, a significant risk factor for transplant rejection and an essential mainspring of mortality in this population. Increased susceptibility to disease and severe infections are due to impairment of the immune system caused by primary diseases and immunosuppressive therapy. Common problems are endogenous infections caused by own microflora. Urogenital mycoplasmas occur in 20–50% of sexually active women. Molecular biology techniques allowed detection of *M. genitalium*, *U. parvum* and *U. urealyticum.* Thanks to this, studies on the epidemiology and etiopathogenesis of urogenital mycoplasmas in human diseases were intensified [1]. Recently in medical literature were published case reports of severe infections caused by urogenital mycoplasmas, very often at atypical localization, especially in patients in risk group for development of opportunistic infections. Furthermore, an important risk factor is also human papillomavirus (HPV). HPV infections can lead to serious consequences and are accepted as an important cause of invasive cervical carcinoma.

In the current study, we assumed the prevalence of urogenital mycoplasmas and HPV among hemodialysed and healthy asymptomatic women.

## 2. Material and Methods 

Examination included 132 sexually active women. The study group consisted of 32 hemodialysed women aged 20–48 (mean age 35.6 ± 8.23 yr) under care of Clinic of Obstetrics and Gynecology, Medical University of Warsaw. The control group included 100 women without diseases and subjectively experienced symptoms from the urogenital tract. The age of the control group was in the range of 20 to 48 years (mean age 33.5 ± 7.49 yr). This study was approved by Bioethical Committee of Medical University of Silesia (KNW/0022/KB1/88/09) and Medical University of Warsaw (KB/117/2007). Exclusion criteria of patients were based on lack of consent, antibiotic therapy and/or chemotherapy and antifungals (at least 4 weeks before examination), pregnancy, diagnosed STI, and vaginal discharge.

First swab from posterior vaginal fornix was taken in order to make a Gram-stained direct smear and evaluated vaginal biocenosis according to the 10-point Nugent score [2].

Second endocervical swab immediately after collection was cultured on Columbia agar with 5% defibrinated sheep blood (for aerobic and anaerobic incubation), Chapman, MacConkey, Sabouraud, de Man, Rogosa, and Sharpe agars. Identification of isolated strains was performed using microbiological analyzer Vitek Compact 2 (bioMérieux, France). Next two endocervical swabs were collected aseptically for determination of urogenital mycoplasmas and HPV, respectively. Culture of *Ureaplasma* spp. and *M. hominis* was performed using Mycoplasma IST2 test (bioMérieux, France), according to manufacturer's instructions.

Isolation of mycoplasmal DNA was performed from pellet of cells obtained from centrifuged (15 000 g, 30 min, at 4°C) mycoplasmal culture using DNeasy Tissue Kit (Qiagen Inc.). Identification of *U. parvum* and *U. urealyticum* was conducted in accordance with Kong et al. [3]. For detection of *M. genitalium* primers for adhesin genes MgPa-1, MgPa-3 and for 16S rRNA gene MG16-45F, MG16-447R, MG16-1204F, and MG16-1301R by Jensen et al. were used [4, 5]. The rule that a double-positive amplicon for adhesin gene with primers MgPa-1/MgPa-3 and double-positive for one of the primers for 16S rRNA gene could be considered as positive was used during interpretation of obtained results. Amplifications were performed using *Taq* PCR Core Kit (Qiagen Inc.) in thermocycler Mastecycler (Eppendorf AG). Negative samples were checked for presence of amplification inhibitors. The reference strains *Ureaplasma urealyticum* ATCC 27618, *Ureaplasma parvum* ATCC 27815 and genomic DNA of *Mycoplasma genitalium* ATCC 33530 were used as positive controls.

Detection of high-risk human papilloma virus (HPV) types 16, 18, 26, 31, 33, 35, 39, 45, 51, 52, 56, 58, 59, 68, 73, 82 and 83 was conducted using Amplicor HPV Test (Roche Molecular System, CA), according to manufacture's instruction.

Obtained results were subjected to statistical analysis in the STATISTICA programme, 9.1.210.0 version (StatSoft Poland, Sp. z o.o.).

## 3. Results

Prevalence of urogenital mycoplasmas in group of hemodialysed women was significantly higher (*P* = 0.0059), compared with the control group, 53.1% and 25%, respectively (Table 1). Furthermore, individual mycoplasmal species were also frequently reported, but these differences were not significant (Table 2).

Taking into consideration the 10-point Nugent score, the majority of the women demonstrated normal vaginal flora. In 18.8% of hemodialysed women versus 4% control group the middle Nugent score was confirmed (4–6). Presence of HPV was detected in 8 women of the study group (25%) and in 23 of the control group (23%) (Tables 3 and 4). Cooccurrence of urogenital mycoplasmas was shown in 6/8 (75%) HPV-positive hemodialysed women and in 7/23 (30.4%) of HPV-positive women in the control group. Among various combinations the most frequent cooccurrence was of HPV and *U. parvum* in the control group and two cases equally in hemodialysed women: HPV + *U. parvum* + *M. hominis* and HPV + *U. urealyticum* (Table 3).

Significant cooccurrence of HPV and mycoplasmas was demonstrated in the control group, in contrast to individual presence of HPV (*P* = 0.0461).

Occurrence of *Lactobacillus* spp., group B *Streptococci* (GBS), Gram-negative rods, and *Candida* spp. was similar in both groups (Table 4). There was no meaningful effect of urogenital mycoplasmas on the prevalence of these microorganisms (data not included). We did not observe any specific symptoms in urogenital tract of women in study and control groups besides presence of urogenital mycoplasmas, HPV, or other potentially pathogenic microorganisms.

## 4. Discussion

Bacterial infections are the second most common cause of death in group of hemodialysed patients [6]. In conducted studies in 27 hemodialysis centres in France, 23% of infections were associated with urinary tract [6, 7]. Among infections with unknown etiology supposed to be also those caused by mycoplasmas. Colonization may constitute a source of dangerous endogenous infections; therefore, occurrence of pathogens in the risk group patients should be monitored. The decrease in the frequency of colonization reduces symptomatic infection rate. In Spanish studies, among a numerous group of renal donors, infections were the direct reason of death in 29% of patients, mainly due to sepsis, pneumonia, and systemic fungal and viral infections [8]. Urogenital mycoplasmas are frequently isolated from clinical materials. Despite the fact that in genitourinary tract they can cause rare infections in immunocompetent patients, but mycoplasmas are an important threat to patients with immunosuppression [9–11]. Yager et al. described a case of peritonitis caused by mycoplasmas in patient with peritoneal dialysis [12].

Majority of positive outcomes in our study concerned *U. parvum* detected in 28% hemodialysed women and in 19% controls and *U. urealyticum* in 15.6% and 5%, respectively. Significant higher percentage of *U. urealyticum* in hemodialyzed women may suggest future possible infections. The absolute domination of *U. parvum* strains (our results) was also shown by other authors [13–19].

After the division of *Ureaplasmas* into two new species *U. parvum* and *U. urealyticum* and demonstration of differences in the frequency of individual species detection, studies were undertaken to determine pathogenicity of these microorganisms. *U. urealyticum* is the commonest etiologic agent of infections, including severe infections in renal transplant patients. *U. urealyticum* detection in patients without infections and subjectively experienced symptoms from the urogenital tract is low (1.2%) [20, 21] and significantly increases in men with NGU [20–22] and women with PID and cervicitis [23]. Besides the fact that we did not note any specific symptoms of cervicitis in hemodialysed women, we speculate that presence of HPV in cervical epithelium may support colonization with* U. urealyticum*. *M. genitalium* DNA detection in our study was 3% in control group and 9.4% in hemodialysed women. Although it is a high percentage, our study was limited by small number of patients in study group. Many studies confirm low percentage of *M. genitalium* in healthy women without symptoms: 4.5% of positive cases were described by English authors [24]. In Denmark, among 731 men and 921 women aged 21–23 without any symptoms in the urogenital tract, *M. genitalium* DNA was demonstrated in 2.3% women and in 1.1% men [25]. *M. genitalium* is clearly defined as an etiologic agent of STI. Clinical indications for next studies of this species are urethritis, epididymitis, and prostatitis in men and cervicitis, urethritis, and PID in women [26]. More than 9% of positive *M. genitalium* cases among studied hemodialysed women suggest importance of this microorganism as an etiological agent of possible complications.

Fairley et al. demonstrated 20% HPV positive cases in hemodialysed women versus 4.5% cases in control group [27]. This suggests that hemodialysed women belong to higher risk group for abnormal Pap and/or development of cervical cancer. In our study we demonstrated equal detection of HPV in hemodialysed and healthy women (25% versus 23%). However, different primers for HPV detection were used by Fairley et al. and in our study (Amplicor detects 13 different genotypes of HPV). We demonstrated cooccurrence of HPV and urogenital mycoplasmas in 75% of hemodialysed women, suggesting importance of this cooccurrence and necessity of further studies.

We summarize that because of demonstrated significant differences in occurrence of urogenital mycoplasmas in hemodialysed women and controls these microorganisms can cause future complications especially in patients preparing for renal transplantation and receiving immunosuppressive therapy.

## 5. Conclusion

Significantly higher incidence of urogenital mycoplasmas and cooccurrence with HPV was demonstrated in hemodialysed women. The need to take into account these microorganisms in routine diagnostics, especially for hemodialysed patient, was demonstrated. Further studies to demonstrate the role of this cooccurrence in etiopathogenesis of infection in hemodialysed patients are required.

## Figures and Tables

**Table 1:** Occurrence of urogenital mycoplasmas in hemodialysed women and the controls (the number and percentage of positive cases).

	Hemodialysed women *n* = 32	Control group *n* = 100
*U. parvum *	6 (18.8%)	16 (16%)
*U. parvum* + *M. hominis *	2 (6.3%)	1 (1%)
*U. parvum* + *M. genitalium *	1 (3.1%)	0
*U. urealyticum *	4 (12.5%)	3 (3%)
*U. urealyticum* + *M. hominis *	1 (3.1%)	0
*M. genitalium *	2 (6.3%)	3 (3%)
*M. hominis *	1 (3.1%)	0
*U. parvum* + *M. hominis* + *U. urealyticum *	0	2 (2%)

Total	17 (53.1%)	25 (25%)

**Table 2:** Frequency of individual urogenital mycoplasmas in the group of hemodialysed women and in controls.

	Study group *n* = 32	Control group *n* = 100	*P*
	Number (%)	
*U. parvum *	9 (28.1)	19 (19)	0.3950
*U. urealyticum *	5 (15.6)	5 (5)	0.0619
*M. genitalium *	3 (9.4)	3 (3)	0.1529
*M. hominis *	4 (12.5)	3 (3)	0.0586

In both groups, *U. parvum* was the most frequent isolate. In the control group this species occurred significantly more often compared with *U. urealyticum* (*P* = 0.0023), *M. genitalium* (*P* = 0.0003), and *M. hominis* (*P* = 0.003). In the group of hemodialysed women *U. parvum* was also the most often detected, however, without statistical significance.

**Table 3:** Cooccurrence of mycoplasmal species in HPV-positive hemodialysed women and control group.

Cooccurrence	HPV-positive hemodialysed women *n* = 8	HPV-positive control group *n* = 23
HPV + *U. parvum *	1	3
HPV* + U. parvum* + *M. hominis *	2	1
HPV + *U. urealyticum *	2	2
HPV + *U. urealyticum* + *M. hominis *	1	0
HPV* + M. genitalium *	0	1
HPV individually	2	16

**Table 4:** Frequency of selected microorganisms in the group of hemodialysed women and controls (the number and percentage of positive cases).

	HPV	*Lactobacillus* spp.	GBS*	*Candida* spp.*	Gram-negative rods*
Hemodialysed women (*n* = 32)	8 (25%)	25 (78.1%)	2 (6.3%)	4 (12.5%)	13 (40.6%)
Control group (*n* = 100)	23 (23%)	87 (87%)	10 (10%)	10 (10%)	23 (23%)

*Single colonies.
